# Coronal decompensation of the trunk by means of a set of show lifts Coronal decompensation of the trunk by means of a set of shoe lifts

**DOI:** 10.1186/1748-7161-8-S2-O30

**Published:** 2013-09-18

**Authors:** Michele Romano, Deborah Luzzi, Alessandra Negrini, Stefano Negrini

**Affiliations:** 1 ISICO, Milan, Italy

## Background

A shoe lift (SL) is often used in the treatment of scoliosis curves in two main cases: (1) an identified discrepancy of the legs' lengths in order to obtain a better balance of the pelvis or (2) a recognized improvement of some specific outcome, like the hump magnitude, when the SL is adopted.

## Purpose

The purpose of this study is to measure the trunk pattern in response to the use of a SL (for this study, a series of SLs). In case of a pre-existing coronal decompensation, we observed the trunk reaction when the SL is respectively under the short leg.

## Methods

We evaluated 27 consecutive patients (26 females and 1 male) who visited our Institute for spine diseases (scoliosis or hyperkyphosis). With the patient in a standing position, we performed a set of tests with a 3-dimensional rastereography (DIERS Formetric 4D) with different SL (5mm, 10mm and 15mm) placed alternatively under each foot. We assessed the variations of two important elements: the change of pelvic inclination and the change of the line that joins C7 and the middle of the sacral spine (C7-MSS). For simplicity, we divided the entire group of patients into two subgroups. One subgroup of patients (13) showed (in standing and normal position) a physiological inclination of the line between C7 and the center of the sacrum towards the right (average 7.08mm ± 6.79). The other subgroup of patients (15) showed a physiological inclination of the line between C7 and the center of the sacrum towards the left (average –12.13mm ± 8.58).

## Results

We found that the use of the SL was not efficient for the improvement of C7-MSS. When the patients showed a physiological inclination of the spine towards the left, we expected a progressive improvement of this inclination if the patients used a SL under the left foot, and a relative worsening if the patients used a SL under the right foot. The situation was the same if the patients had a physiological inclination of this line towards the right. The results show a completely different pattern. Tables 1 and 2 below show the mean inclinations of this line, using the three SL:

**Table 1:** 

Physiological inclination of the spine towards the **right** (13 patients)					
**S.L.under the right foot** and relative inclinations					

S.L. 5mm	**6.62±6.5**	S.L. 10mm	**6.77±11.12**	S.L. 15mm	**7.38 ±8.18**

**S.L. under the left foot** and relative inclinations					

S.L. 5mm	**6.92±9.05**	S.L. 10mm	**8,54 ±9.29**	S.L. 15mm	**5.92±7.19**

**Table 2:** 

Physiological inclination of the spine towards the **left** (15 patients)					
**S.L. under the left foot** and relative inclinations					

S.L. 5mm	**-11.27±10.91**	S.L. 10mm	**-13.07±11.49**	S.L. 15mm	**-13.00±17.30**

**S.L. under the right foot** and relative inclinations					

S.L. 5mm	**-10.40±8.43**	S.L. 10mm	**-10.80±8.24**	S.L. 15mm	**-9.47±14.68**

## Conclusions and discussion

The use of a SL (5mm, 10mm and 15mm) positioned under each foot did not change the pattern of the inclination (theoretically, a reduction or an increase of the inclination of C7-MSS) measured in standing and normal positions. The spine seems to have an individual anti-gravity pattern that cannot be modified by the use of a SL.

